# Economic analysis of pandemic influenza mitigation strategies for five pandemic severity categories

**DOI:** 10.1186/1471-2458-13-211

**Published:** 2013-03-08

**Authors:** Joel K Kelso, Nilimesh Halder, Maarten J Postma, George J Milne

**Affiliations:** 1 School of Computer Science and Software Engineering, University of Western Australia, Perth, Western Australia, Australia; 2 Unit of PharmacoEpidemiology and PharmacoEconomics, Department of Pharmacy, University of Groningen, Groningen, Netherlands

**Keywords:** Pandemic influenza, Economic analysis, Antiviral medication, Social distancing, Pandemic severity, Case fatality ratio

## Abstract

**Background:**

The threat of emergence of a human-to-human transmissible strain of highly pathogenic influenza A(H5N1) is very real, and is reinforced by recent results showing that genetically modified A(H5N1) may be readily transmitted between ferrets. Public health authorities are hesitant in introducing social distancing interventions due to societal disruption and productivity losses. This study estimates the effectiveness and total cost (from a societal perspective, with a lifespan time horizon) of a comprehensive range of social distancing and antiviral drug strategies, under a range of pandemic severity categories.

**Methods:**

An economic analysis was conducted using a simulation model of a community of ~30,000 in Australia. Data from the 2009 pandemic was used to derive relationships between the Case Fatality Rate (CFR) and hospitalization rates for each of five pandemic severity categories, with CFR ranging from 0.1% to 2.5%.

**Results:**

For a pandemic with basic reproduction number R_0_ = 1.8, adopting no interventions resulted in total costs ranging from $441 per person for a pandemic at category 1 (CFR 0.1%) to $8,550 per person at category 5 (CFR 2.5%). For severe pandemics of category 3 (CFR 0.75%) and greater, a strategy combining antiviral treatment and prophylaxis, extended school closure and community contact reduction resulted in the lowest total cost of any strategy, costing $1,584 per person at category 5. This strategy was highly effective, reducing the attack rate to 5%. With low severity pandemics costs are dominated by productivity losses due to illness and social distancing interventions, whereas higher severity pandemic costs are dominated by healthcare costs and costs arising from productivity losses due to death.

**Conclusions:**

For pandemics in high severity categories the strategies with the lowest total cost to society involve rigorous, sustained social distancing, which are considered unacceptable for low severity pandemics due to societal disruption and cost.

## Background

While the H1N1 2009 virus spread world-wide and was classed as a pandemic, the severity of resulting symptoms, as quantified by morbidity and mortality rates, was lower than that which had previously occurred in many seasonal epidemics [1-3]. The 2009 pandemic thus highlighted a further factor which must be considered when determining which public health intervention strategies to recommend, namely the severity of symptoms arising from a given emergent influenza strain. The mild symptoms of H1N1 2009 resulted in a reluctance of public health authorities to use rigorous social distancing interventions due to their disruptive effects, even though modelling has previously suggested that they could be highly effective in reducing the illness attack rate [4-13].

Had the H1N1 2009 influenza strain been highly pathogenic, more timely and rigorous responses would have been necessary to mitigate the resultant adverse health outcomes. Furthermore, there is continuing concern that a highly pathogenic avian influenza A(H5N1) strain may become transmissible between humans. This scenario is highlighted by the large reservoir of influenza A(H5N1) in poultry in South-East Asia [14], and recent experimental results which have shown that the A(H5N1) virus may be genetically modified to become readily transmissible between ferrets, a commonly used animal model for human influenza transmission studies [15-17].

The severity of a particular influenza strain directly impacts on the cost of any pandemic; increased severity increases health care costs and escalates productivity losses due to *a)* absenteeism arising from increased illness and *b)* increased mortality rates. In this study, the role which pandemic severity has on the total cost of a pandemic for a range of potential intervention strategies is analysed, and for highly pathogenic influenza strains inducing significant morbidity and mortality, as occurred during the 1918 pandemic [18,19], the results suggest which intervention strategies are warranted in terms of reduction of illness and total pandemic cost. This study adopts a societal perspective on the cost of a pandemic, with the time horizon being the lifetime of individuals experiencing the pandemic.

## Methods

### General overview

**Figure 1:**
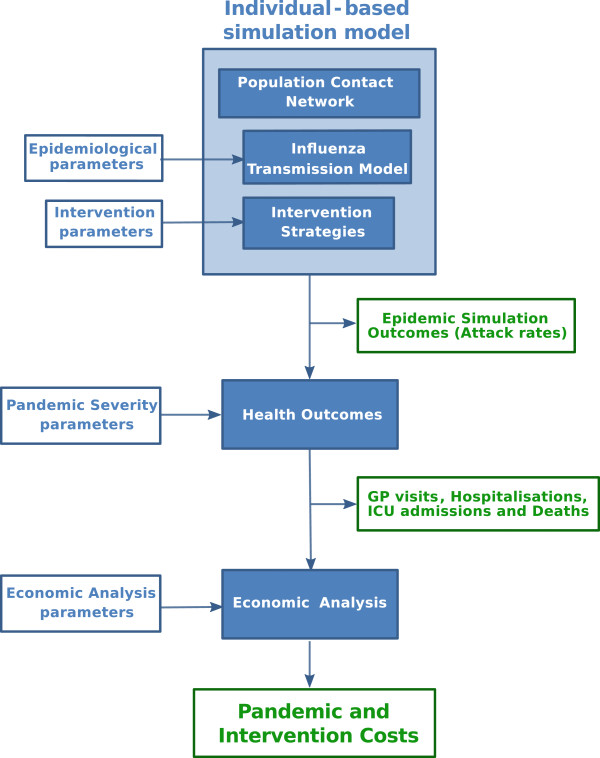
**Overview of pandemic cost analysis methodology.** Input parameters are shown on the left in boxes with blue text, with arrows indicating to which part of the cost analysis methodology they apply. Boxes with white text represent different processes of the methodology – each process is described in the Methods section under a subsection of the same name. Boxes with green text appearing at the bottom and on the right represent results generated by the analysis.

We used a detailed, individual-based simulation model of a real community in the south-west of Western Australia, the town of Albany with a population of approximately 30,000, to simulate the dynamics of an influenza pandemic. Comparing simulations with and without interventions in place allowed us to analyse the effect which a range of interventions have on reducing the attack rate and on the health of each individual in the modelled community. Epidemic outcome data produced by the simulation model were used to determine health outcomes involving hospitalisation, ICU treatment, and death. In turn, these healthcare outcomes, together with productivity losses due to removal from the workforce, were used to estimate the overall cost of interventions. Figure 1 provides an overview of this analysis methodology, showing each of the processes that make up the methodology, their input parameters and the resulting data generated by the process.

### Simulation model

#### Population contact network

The simulation model captures the contact dynamics of the population of Albany, Western Australia using census and state and local government data [20]. These data allowed us to replicate the individual age and household structure of all households in this town of approximately 30,000 individuals, and also allowed for the construction of an explicit contact network linking households, schools, workplaces and other meeting places by allocating individuals to workplaces and schools.

**Figure 2:**
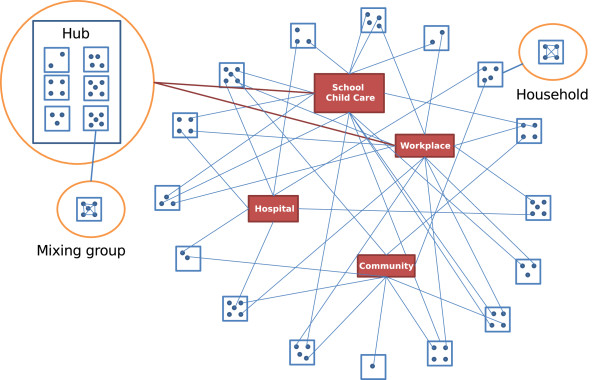
Idealised household and hub contact network.

The modelled community was chosen so as to be representative of a developed world population, and self-contained in the sense that all major locales for interpersonal mixing were represented within the community. The model includes both urban and rural components, a central commercial core, a complete set of schools (covering all age groups), and a mix of large and small employers. The community is also of a size where public health interventions could be uniformly implemented based on local information. The model captures explicit person-to-person contact with the contact network describing population mobility occurring between households, schools, workplaces and the wider community as shown in Figure 2. The virus spreads through the community due to this mobility, as transmission occurs between individuals when they are co-located, possibly following a move from one location to another. For example, an infectious child moves from household to school on a given day, and infects two further children; they return to households 2 and 3 and, following virus incubation, become infectious and may infect other household members in their households. Note that these households may be geographically separate, but are connected via contact of children at school.

Each household contains uniquely identified individuals. Children and adults were assigned by an allocation algorithm to school classes and workplaces, respectively. The assignment of children to classes was based on age, school class size data, and proximity between schools and households; the assignment of adults to workplaces was based on workplace size and commuter survey data. In addition to contact occurring in households and mixing hubs, community contact was introduced to capture mixing which occurs outwith these locales and in the wider community.

The number of contacts made by each individual each day in school, work and community settings were adjusted to reproduce the proportion of cases occurring in different settings as reported by empirical studies, specifically 40% of infections occurred in households, 30% in schools and workplaces, and 30% in the wider community [21-23]. Contacts within schools and workplaces occurred in fixed-size mixing groups of maximum size 10. Within mixing groups contact was assumed to be homogeneous. Community contacts occurred between randomly selected individuals, weighted toward pairs of individuals located in neighbouring households.

#### Epidemic simulations

A simulation algorithm, realised in the C++ programming language, manipulates the underlying demographic model and captures both population mobility and the time-changing infectivity profile of each individual. Each individual has their infectivity status denoted by one of the four (Susceptible, Exposed, Infectious, Recovered) states at any time point during the duration of the simulated period. The simulation algorithm captures the state of the whole population twice per day, a daytime point-in-time snapshot and an evening snapshot, with individuals (possibly) moving locations between successive day or night periods, such as household to school or workplace for the day phase, returning to home for the night period. Individuals come into contact with other individuals on a one-to-one basis in each location, with possible influenza transmission then occurring. Individuals in each household and contact hub make contacts within a close-contact mixing group, taken to be the entire household or a subset of larger hubs, and also make additional non hub-based random community contacts. The attributes of the various locations in which individuals come into potentially infectious contact are summarized in Table 1.

**Table 1:** Individual and contact location attributes

**Agents/Objects**	**Description**			
***Individual***	The individual is the primary agent entity in the model. Each individual has an age, a household, a (possible) daytime hub, an activity state, a current infection state, and time-of-infection (when infected) that explicitly tracks the progression of the infectious disease. Individuals move between different locations at different time periods, making contacts with other individuals.			
**Locations**	**Description**	**When contact occurs**	**Who participates in contact**	**Average number of contacts**
***Household***	Each household is made up from a certain number of individuals following census data, spatially located.	Every day during Day and Night cycle.	Members of each household	Average household size is 2.54 individual
***Childcare***	Child care and pre-schools; number and size determined from local government data.	Weekdays during Day cycle.	Child (age 0–4) and adult (worker) individuals who are allocated into the hub if they are active*.	Maximum group size is 10.
***School***	Primary and secondary schools; number, size and age structure determined from state education department data.	Weekdays during Day cycle.	Child (ages 5–17) and adult (teacher) individuals who are allocated into the hub if they are active*.	Maximum group size is 10.
***Adult education***	Tertiary and vocational education institutions, number and size determined from state education department data.	Weekdays during Day cycle.	Young adult and adult individuals who are allocated into the hub if they are active*.	Maximum group size is 10.
***Workplace***	Number and size of determined for local government business survey data.	Weekdays during Day cycle.	Adult individuals who are allocated into the hub if they are active^*^.	Maximum group size is 10.
***Community***	Represents all contact between individuals in the community that is not repeated on a daily basis.	Everyday during Day cycle.	All individuals make contacts if they are active*, contact is random but weighted towards pairs with nearby household locations.	4 contacts for each individual (2 if community contact reduction is in effect)

* All individuals are active during day cycles unless: he/she is symptomatically infected and chooses to withdraw to household (50% chance for adults, 90% for children); or if his/her school or workplace is affected by social distancing interventions; or if he/she is a parent of a child who is inactive (only one parent per family is affected this way).

Using the contact, mobility and transmission features described above, stochastic simulations of influenza spread were conducted. All simulations were repeated 40 times with random numbers controlling the outcome of stochastic events (the locality of seeded infected individuals and the probability of transmission) and the results were averaged. Analysis of this simulation model has shown that the 40-run mean attack rate is highly unlikely (95% confidence) to differ by more than 1.2% from the mean attack rate of a much larger set of experiment repeats.

One new infection per day was introduced into the population during the whole period of the simulations, and randomly allocated to a household. This seeding assumption of 1 case per day was chosen to reliably begin a local epidemic in every stochastic simulation. For the transmission characteristics described above, analysis shows that seeding at this rate for 7 days results in a sustained epidemic in >97% of the simulation runs and 100% with two weeks of seeding, with higher percentages for the higher transmissibility scenarios. Seeding at this rate is continued throughout the simulation in order to capture the case where an epidemic may be initially suppressed by a rigorous intervention strategy, but may subsequently break out if intervention measures are relaxed.

After the beginning of a sustained local epidemic, any subsequent variation in the amount of seeding has very little effect on the progress of the local epidemic, as the number of imported cases is much smaller than those generated by the local epidemic. Preliminary analyses using the present model have shown that even if the seeding rate is increased to 5 infections per day, after 7 days the number of infections generated from the self-sustained local epidemic is twice the number of imported infections, and by 14 days local infections outnumber imported infections by a factor of 8.

The simulation period was divided into 12 hour day/night periods and during each period a nominal location for each individual was determined. This took into consideration the cycle type (day/night, weekday/weekend), infection state of each individual and whether child supervision was needed to look after a child at home. Individuals occupying the same location during the same time period were assumed to come into potential infective contact. Details of the simulation procedure are presented in [10].

#### Influenza transmisison model

In the simulation model, we assumed that infectious transmission could occur when an infectious and susceptible individual came into contact during a simulation cycle. Following each contact a new infection state for the susceptible individual (either to remain susceptible or to become infected) was randomly chosen via a Bernoulli trail [24]. Once infected, an individual progressed through a series of infection states according to a fixed timeline.

The probability that a susceptible individual would be infected by an infectious individual was calculated according to the following transmission function, which takes into account the disease infectivity of the infectious individual *I*_*i*_ and the susceptibility of susceptible individual *I*_*s*_ at the time of contact.

$$P_{\mathrm{trans}}\left(I_{i},I_{s}\right)=\beta \times \mathrm{Inf}\left(I_{i}\right)\times \mathrm{Susc}\left(I_{s}\right)\times \mathrm{AVF}\left(I_{i},I_{s}\right)$$

The baseline transmission coefficient β was initially chosen to give an epidemic with a final attack rate of 17.4%, which is consistent with seasonal influenza as estimated in [25] (in Table three of that paper). To achieve simulations under a range of basic reproduction numbers (R_0_), β was increased from this baseline value to achieve epidemics of various R_0_ magnitudes; details of the procedure for estimating β and R_0_ are given in [10]. A reproduction number of 1.8 was used as a baseline assumption, and the sensitivity of results to this assumption was gauged by repeating all simulations and analyses for alternative reproduction numbers of 1.5 and 2.5. A pandemic with a reproduction number of 1.5 corresponds to some estimations of the basic reproduction number of the 2009 pandemic [26-29], while a reproduction number of 2.5 corresponds to an upper bound on estimates of what may have occurred in the 1918 pandemic, with most estimates being in the range 1.8-2.2 [18,19].

The disease infectivity parameter *Inf*(*I*_*i*_) was set to 1 for symptomatic individuals at the peak period of infection and then to 0.5 for the rest of the infectivity period. The infectiousness of asymptomatic individuals was also assumed to be 0.5 and this applies to all infected individuals after the latent period but before onset of symptoms. The infection profile of a *symptomatic* individual was assumed to last for 6 days as follows: a 0.5 day latent period (with *Inf*(*I*_*i*_) set to 0) was followed by 1 day asymptomatic and infectious, where *Inf*(*I*_*i*_) is set to 0.5; then 2 days at peak infectiousness (with *Inf*(*I*_*i*_) set to 1.0); followed by 2.5 days reduced infectiousness (with *Inf*(*I*_*i*_)set to 0.5). For an infected but *asymptomatic* individual the whole infectious period (of 5.5 days) was at the reduced level of infectiousness with *Inf*(*I*_*i*_) set to 0.5. This infectivity profile is a simplification of the infectivity distribution found in a study of viral shedding [30]. As reported below in the results section for the unmitigated no intervention scenario, these assumptions regarding the duration of latent and infectious periods lead to a mean generation time (serial interval) of 2.47 days which is consistent with that estimated for H1N1 2009 influenza [26,31,32].

Following infection an individual was assumed to be immune to re-infection for the duration of the simulation. We further assume that influenza symptoms developed one day into the infectious period [30], with 20% of infections being asymptomatic among children and 32% being asymptomatic among adults. These percentages were derived by summing the age-specific antibody titres determined in [33]. Symptomatic individuals withdrew into the home with the following probabilities; adults 50% and children 90%, which is in keeping with the work of [23,34].

The susceptibility parameter *Susc*(*I*_*s*_) is a function directly dependent on the age of the susceptible individual. It captures age-varying susceptibility to transmission due to either partial prior immunity or age-related differences in contact behaviour. To achieve a realistic age specific infection rate, the age-specific susceptibility parameters were calibrated against the serologic infection rates for seasonal H3N2 in 1977–1978 in Tecumseh, Michigan [25]. The resulting age-specific attack rates are consistent with typical seasonal influenz, with a higher attack rate in children and young adults (details of the calibration procedure may be found in [10]).

The antiviral efficacy factor *AVF*(*I*_*i*_,*I*_*s*_) = (1 - *AVE*_*i*_)*(1 - *AVE*_*s*_) represents the potential reduction in infectiousness of an infected individual (denoted by *AVE*_*i*_) induced by antiviral treatment, and the reduction in susceptibility of a susceptible individual (denoted by *AVE*_*s*_) induced by antiviral prophylaxis. When no antiviral intervention was administrated the values of both *AVE*_*i*_ and *AVE*_*s*_ were assumed to be 0, indicating no reduction in infectiousness or susceptibility. However, when antiviral treatment was being applied to the infectious individual the value of *AVE*_*i*_ was set at 0.66, capturing a reduction in infectiousness by a factor of 66% [35]. Similarly, when the susceptible individual was undergoing antiviral prophylaxis the value of *AVE*_*s*_ was set to 0.85 indicating a reduction in susceptibility by a factor of 85% [35]. This estimate is higher than most previous modelling studies [6,36,37], which assume an *AVE*_*s*_ of 30%. This common assumption appears to stem from an estimate made in [38] based on 1998–1999 trial data. Our higher value is based on a more comprehensive estimation process reported in [35], which also incorporated data from an additional study performed in 2000–2001 [39]. It is also in line with estimates of 64%-89% reported in [40].

#### Intervention strategies

We examined a comprehensive range of intervention strategies including school closure, antiviral drugs for treatment and prophylaxis, workplace non-attendance (workforce reduction) and community contact reduction. These interventions were considered individually and in combination and social distancing interventions were considered for either continuous periods (that is, until the local epidemic effectively ceased) or periods of fixed duration (2 weeks or 8 weeks).

Antiviral drug interventions and social distancing interventions were initiated when specific threshold numbers of symptomatic individuals were diagnosed in the community, and this triggered health authorities to mandate the intervention response. This threshold was taken to be 0.1% of the population. This threshold was chosen based on a previous study with this simulation model, which found that it represents a robust compromise between early, effective intervention and “premature” intervention, which can result in sub-optimal outcomes when limited duration interventions are used [5]. It was assumed that 50% of all symptomatic individuals were diagnosed, and that this diagnosis occurred at the time symptoms appeared.

For continuous school closure, all schools were closed simultaneously once the intervention trigger threshold was reached. For fixed duration (e.g. 2 weeks or 8 weeks) school closure, schools were closed individually as follows: for a primary school the whole school was closed if 1 or more cases were detected in the school; in a high school only the class members of the affected class were isolated (sent home and isolated at home) if no more than 2 cases were diagnosed in a single class; however if there were more than 2 cases diagnosed in the entire high school the school was closed. Note that these school closure policies were only activated after the community-wide diagnosed case threshold was reached; cases occurring in schools before this time did not result in school closure. This policy of triggering school closure based on epidemic progression avoids premature school closure which can reduce the effectiveness of limited duration school closure [5,36,41]; see [5] for a detailed description of school closure initiation triggering strategies.

Two primary antiviral drug strategies have been examined; antiviral drugs used solely for treatment of symptomatic cases (strategy T), and treatment plus prophylaxis of all household members of a symptomatic case (strategy AV). A further strategy was also examined, in which prophylaxis was also extended to the contact group (school or workplace contacts) of a symptomatic case (strategy T + H + E). Due to the logistical resources required, it is unlikely that this extended strategy could be implemented throughout a pandemic, and we do not report the results of this strategy in the main paper; full results are however given in (Additional file 1). Antiviral treatment (and prophylaxis for household or work / school group contacts) was assumed to begin 24 hours after the individual became symptomatic. It was assumed that an individual would receive at most one prophylactic course of antiviral drugs. Further details of antiviral interventions are given in [4,37].

Workforce reduction (WR) was modelled by assuming that for each day the intervention was in effect, each worker had a 50% probability of staying at home and thus did not make contact with co-workers. Community contact reduction (CCR) was modelled by assuming that on days when the intervention was in effect, all individuals made 50% fewer random community contacts. The most rigorous social distancing interventions considered in this study, which we denote as strict social distancing, involve the combined activation of school closure with workforce reduction and/or community contact reduction, and for this to occur for significant time periods; continuous and 8 weeks duration were considered.

In the present study we simulated a total of 32 intervention scenarios (for each of three reproduction numbers 1.5, 1.8 and 2.5). To simplify the results, we only present those interventions that reduce the unmitigated illness attack rate by at least 50%.

### Definition of severity

We defined five severity categories based on those proposed by the CDC [42]. The CDC pandemic index was designed to better forecast the health impact of a pandemic, based on 5 categories having CFRs ranging from <0.1% to > = 2.0%, and allow intervention recommendations to match pandemic severity. The discrete CFRs used are listed in Table 2. We extend the CDC categories to further include rates of hospitalisation and ICU treatment, as described below using data collected during the 2009 pandemic in Western Australia, by the state Department of Health. These data permit case hospitalisation (ICU and non-ICU) and case fatality ratios (CFR) to be related, as described below.

**Table 2:** Model parameters and cost data

**Parameters**	**Values**	**Source**
**Epidemiological parameters**		
Symptomatic infectiousness timeline	0.5 day latent (non infectious), 1 day asymptomatic; 2 days peak symptomatic; 2.5 days post-peak symptomatic	[30]
Asymptomatic infectiousness timeline	0.5 day latent; 5.5 days asymptomatic	[30]
Asymptomatic infectiousness	0.5	[30]
peak symptomatic infectiousness	1.0	-
post-peak symptomatic infectiousness	0.5	[30]
Probability of asymptomatic infection	0.32	[33]
Probability of adult withdrawal from work if sick	0.5 (0.25,0.75)*	-
Probability of child withdrawal from school if sick	0.9 (0.5,1.0)*	-
Basic Reproduction Number R_0_ and Attack Rate (%)	R_0_ = 1.5, AR = 24.4% (R_0_ = 1.8 and AR = 32.4%, R_0_ = 2.5, AR = 43.8%)*	-
**Intervention parameters**		
Antiviral infectiousness reduction	66% (33%, 89%)*	[35,40]
Antiviral susceptibility reduction	85% (43%, 90%)*	[35,40]
Prophylaxis symptom reduction probability	50%	[35]
Diagnosis delay	12 h	-
Diagnosis ratio	50%	-
Maximum antiviral courses given for treatment	1 course per person for 5 days	-
Maximum antiviral courses given for prophylaxis	1 course per person for 10 days	-
School Closure Duration	2 weeks, 8 weeks and continuously	-
School Closure Trigger	20 to 40 community cases	[5]
School Closure withdrawal probability	1.0 (0.5, 0.75)*	-
Workforce Reduction Duration	4 weeks and continuously	-
Workforce Reduction Trigger	2 weeks after first case	-
Workforce Reduction attendance probability	0.5 (0.25, 0.75)*	-
Community Contact Reduction (CCR) Duration	4 weeks and continuously	-
CCR Trigger	2 weeks after first case	-
CCR withdrawal probability	0.5 (0.25, 0.75)*	-
**Pandemic severity parameters**		
Severity Category 1 (CFR < 0.1%)	**Case Fatality Rate** = 0.1%	[42]
Severity Category 2 (CFR 0.1% - 0.5%)	**Case Fatality Rate** = 0.25%	[42]
Severity Category 3 (CFR 0.5% - 1.0%)	**Case Fatality Rate** = 0.75%	[42]
Severity Category 4 (CFR 1.0% - 2.0%)	**Case Fatality Rate** = 1.5%	[42]
Severity Category 5 (CFR > = 2.0%)	**Case Fatality Rate** = 2.5%	[42]
Hospitalisation / fatality ratio	32:1	
ICU / fatality ratio	3:1	
Average hospital stay (days)	4	[1,43]
Average ICU stay (days)	7	[1,43]
**Economic analysis parameters**		
Average wages (per week)	$836	[44]
Average school closure cost (per student per day)	$19.22	[45]
Average GP visit cost	$106.97	[46]
Average hospitalization cost (per day)	$1042	[46]
Average ICU cost (per day)	$2084	[46,47]
Antiviral cost per course	$24.81	[46]
Antiviral dispensing cost per course	$31.22	[46]
Antiviral shelf life	5 years	[48]
Mean time between pandemics	30.3 years	-
Discount Rate (annually)	3%	[49]

* indicates alternative values analysed in sensitivity analyses.

The least severe pandemic considered (category 1) has CFR of 0.1% which is at the upper end of estimates for the 2009 pandemic. Initially, the 2009 pandemic CFR was estimated to be in the range 0.007% - 0.048% [50]; however recent reanalysis of global data from 2009 suggest a CFR (for the 18–64 age group) in the range 0.018% - 0.159% [51]. Cost analysis results for a pandemic with H1N1 2009 characteristics using a similar simulation model to the one described here can be found in [52].

### Health outcomes

Calculation of costs arising from lost productivity due to death and from hospitalisation of ill individuals requires that individual health outcomes (symptomatic illness, hospitalisation, ICU admission, and death) be estimated for each severity level. The 2009 pandemic data from Western Australia was used to provide this relationship between the mortality rate and numbers requiring hospitalisation and ICU care. These data indicated a non-ICU hospitalisation to fatality ratio of 32:1 and an ICU admission to fatality ratio of 3:1. These values align with those in a previous study by Presanis et al. in [50], which estimated the ratios in the ranges 17–37 to 1 and 3.1–5.0 to 1, respectively.

### Economic analysis

The economic analysis model translates the age-specific infection profile of each individual in the modelled population, as derived by the Albany simulation model, into the overall pandemic cost burden. Total costs involve both direct healthcare costs (e.g. the cost of medical attention due to a GP visit, or for hospitalisation) and costs due to productivity loss [47,53]. Pharmaceutical costs (i.e. costs related to antiviral drugs) are also estimated. All costs are reported in 2010 US dollars using consumer price index adjustments [54]. 2010 US dollar values are used to make the results readily convertible to a wide range of countries.

Age-specific hospitalisation costs are achieved by multiplying the average cost per day by average length of stay for each age group [55,56]. Hospitalisation costs, including ICU costs, those involving medical practitioner visits, and antiviral drug (and their administration) costs are taken from the literature and are presented in Table 2[46,47,53]. The antiviral costs include the costs of maintaining an antiviral stockpile. This was calculated by multiplying the antiviral cost per course (but not the dispensing cost per course, which was included separately) by the expected number of times each antiviral course would expire and be replaced between pandemics, assuming a mean inter-pandemic period of 30.3 years (based on the occurrence of pandemics in 1918, 1957, 1968 and 2009) and an antiviral shelf life of 5 years [48]. Treatment costs, lengths of stay in hospital (both ICU and non-ICU), and other cost data used in establishing the overall cost of mitigated and unmitigated epidemics in the modelled community are given in Table 2.

Productivity losses due to illness and interventions (e.g. necessary child-care due to school closure and workforce reduction) were calculated according to the human capital approach, using average wages and average work-days lost; the latter being determined from day-to-day outbreak data generated by the simulation model. Assumed average wages are given in Table 2.

School closure is assumed to give rise to two costs. The first, following the work of Perlroth et al. [45], is a $19 per student school day lost. This is intended to approximate the cost of additional education expense incurred in the future – which might occur for example in the form of additional holiday classes. The second component is lost productivity of parents staying at home to supervise children. The simulation model calculates whether this occurs for every day for every household, based on what interventions are in force (school closure and/or workforce reductions), whether children or adults are ill, the number of adults in the household, whether it is a school day, etc., and accumulates the cost accordingly.

Indirect production losses due to death were also derived using a human capital approach, based on the net present value of future earnings for an average age person in each age group. This was calculated by multiplying the age-specific number of deaths due to illness by the average expectancy in years of future earnings of an individual by an average annual income [44]. We assumed a maximum earning period up to age 65. Productivity losses due to death were discounted at 3% annually, which is a standard discounting rate used to express future income in present value [49]. To provide an alternative analysis, total costs were also calculated without this long-term productivity loss due to death component.

## Results

### Overview

**Figure 3:**
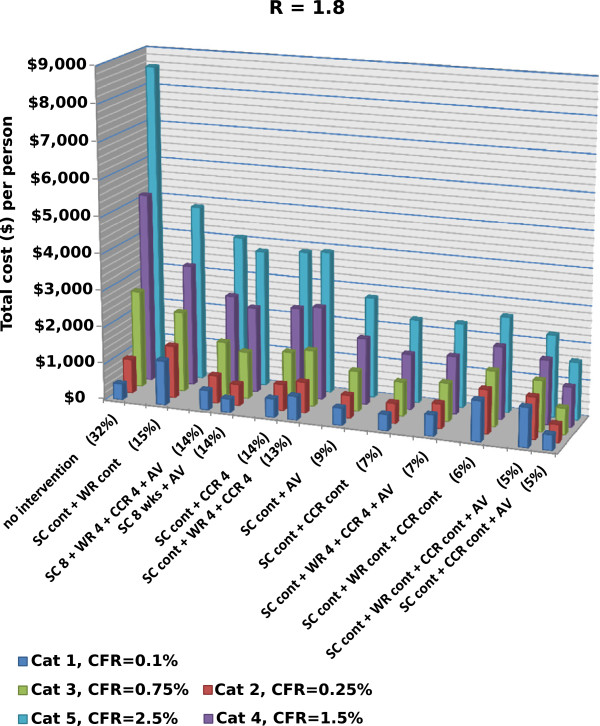
**Total cost of intervention strategies for 5 pandemic severity categories.** Total pandemic cost for each severity category. Costs shown by colour coded columns according to pandemic severity, with cost per person in community shown on left axis. Intervention strategies are listed on horizontal axis. Attack rates (AR) for each strategy appear with each strategy label. Values are for a pandemic with unmitigated transmissibility of R_0_ = 1.8. Interventions abbreviated as: SC – school closure; CCR – 50% community contact reduction; WR – 50% workforce reduction; 4, 8 – intervention duration in weeks; cont – continuous duration; AV – antiviral treatment of diagnosed symptomatic cases and antiviral prophylaxis of household members of diagnosed symptomatic cases.

Figure 3 presents the final attack rate (AR) and the total cost of the epidemic for each intervention strategy applied, for a pandemic with a basic reproduction number of R_0_ = 1.8. Although costs are calculated from the whole-of-society perspective, total costs are presented as a cost per person in the community, calculated by dividing the simulated cost of the pandemic by the population of ~30,000, in order to make the results more easily transferable to communities of various sizes. Strategies are ordered from left to right by increasing effectiveness (i.e. their ability to decrease the attack rate), and only intervention strategies that reduce the attack rate by at least 50% are included.

Figure 3 shows three distinctive features. Firstly, for an epidemic with basic reproduction number R_0_ = 1.8, no single intervention is effective in reducing the attack rate by more than 50%, and thus do not appear in Figure 3. This finding is consistent with previous modelling studies which found that layering of multiple interventions is necessary to achieve substantial attack rate reductions [5-10,12,57,58]. Secondly, higher severity pandemics have higher total costs. Total costs of unmitigated pandemics range from $441 to $8550 per person for pandemics from category 1 to category 5 (see Table 3). Thirdly, for high severity pandemics total costs are lower for the more effective intervention strategies.

**Table 3:** Intervention total costs

**Intervention strategy**	**AR (%)**	**Cat 1**	**Cat 2**	**Cat 3**	**Cat 4**	**Cat 5**
		**Case Fatality Ratio (CFR)**				
		**0.1%**	**0.25%**	**0.75%**	**1.5%**	**2.5%**
no intervention	32.4	$441	$943	$2649	$5175	$8550
*SC cont + WR cont	15.7	$1217	$1439	$2194	$3311	$4804
SC 8 + WR 4 + CCR 4 + AV	14.9	$539	$757	$1499	$2596	$4062
SC 8 wks + AV	14.5	$374	$582	$1288	$2334	$3732
*SC cont + CCR 4	14.5	$518	$722	$1419	$2449	$3826
*SC cont + WR 4 + CCR 4	13.2	$654	$854	$1533	$2539	$3882
SC cont + AV	9.2	$489	$629	$1104	$1808	$2748
*SC cont + CCR cont	7.4	$447	$560	$945	$1514	$2275
SC cont + WR 4 + CCR 4 + AV	7.9	$585	$691	$1052	$1585	$2298
*SC cont + WR cont + CCR cont	6.0	$1116	$1208	$1521	$1984	$2603
SC cont + CCR cont + AV	5.7	$416	$488	$734	$1098	$1584
SC cont + WR cont + CCR cont + AV	5.6	$1083	$1155	$1401	$1764	$2249

Cost of pandemic shown as total cost for each intervention strategy and each severity category. Costs expressed as dollars (US) per member of population. Values are for pandemic with unmitigated transmissibility of R_0_ = 1.8. Interventions abbreviated as: SC – school closure; CCR – 50% community contact reduction; WR – 50% workforce reduction; 4, 8 – intervention duration in weeks; cont – continuous duration; AV – antiviral treatment of diagnosed symptomatic cases and antiviral prophylaxis of household members of diagnosed symptomatic cases. Purely social distancing interventions marked by *.

**Figure 4:**
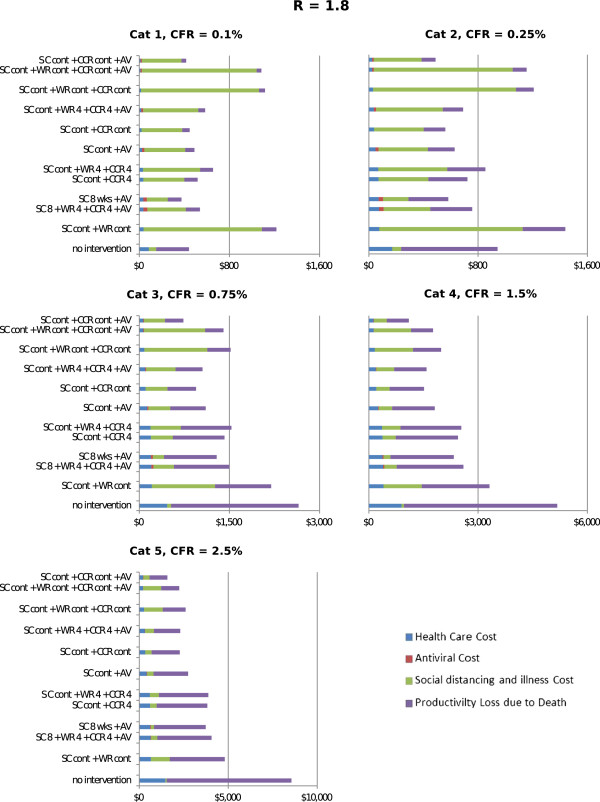
**Breakdown of pandemic cost components.** Breakdown of pandemic costs shown as horizontal bar, for each intervention strategy and each severity category. Coloured segments of each bar represent cost components as follows: (blue) health care; (red) antiviral drugs, including dispensing costs; (green) productivity losses due to illness and social distancing interventions; (purple) productivity losses due to deaths. Note that horizontal scale is different for each severity category. Values are for a pandemic with unmitigated transmissibility of R_0_ = 1.8. Interventions abbreviated as: SC – school closure; CCR – 50% community contact reduction; WR – 50% workforce reduction; 4, 8 – intervention duration in weeks; cont – continuous duration; AV – antiviral treatment of diagnosed symptomatic cases and antiviral prophylaxis of household members of diagnosed symptomatic cases.

Figure 4 presents the constituent components that make up the total cost of each intervention and severity category, measured in terms of cost per person in the modelled community. Three distinctive features can be seen in Figure 4. Firstly, for high severity pandemics costs are dominated by productivity losses due to death and health care costs. Secondly, for low severity pandemics costs are dominated by social distancing and illness costs. Thirdly, for all severity categories antiviral costs are comparatively low when compared with all other cost components of antiviral based intervention strategies. Antiviral costs never constitute more than 20% of the total cost, and for all severity categories greater than 1 (CFR >0.1%) antiviral costs are always the smallest cost component.

Below we report on effectiveness, total costs and cost components of interventions for pandemics with high and low severity. These cost data are presented in Table 3.

### High severity pandemics

**Figure 5:**
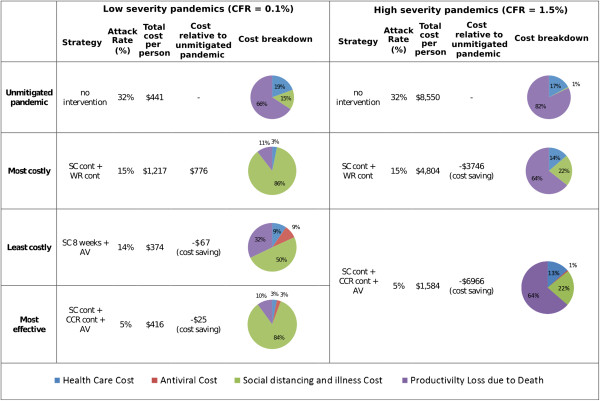
**Summary of key intervention strategies.** Characteristics of key intervention strategies is given for pandemics of low severity (category 1, CFR < = 0.1%) and high severity (category 5, CFR > = 2.5%). Values are for a pandemic with unmitigated transmissibility of R_0_ = 1.8. Interventions abbreviated as: SC – school closure; CCR – 50% community contact reduction; WR – 50% workforce reduction; 4, 8 – intervention duration in weeks; cont – continuous duration; AV – antiviral treatment of diagnosed symptomatic cases and antiviral prophylaxis of household members of diagnosed symptomatic cases.

Figure 5 summarises the characteristics of key intervention strategies. For high severity pandemics (categories 4 and 5, with case fatality rates above 1.5%) the least costly strategy combines continuous school closure, community contact reduction, antiviral treatment and antiviral prophylaxis. At category 5 this strategy has a total cost of $1,584 per person, a net benefit of $6996 per person compared to no intervention. This strategy is also the most effective intervention strategy, reducing the attack rate from 32% to 4.6%. The results indicate that strategies with the lowest total costs are also the most effective. For a category 5 pandemic the 6 most effective strategies, all of which reduce the attack rate to less than 10%, have total costs ranging from $1,584 to $2,748 per person, which is less than one-third the cost of the unmitigated pandemic ($8,550), showing the substantial net benefit of effective interventions for high severity pandemics. These strategies all feature continuous school closure, with either continuous community contact reduction or antiviral treatment and prophylaxis.

The ability of highly effective interventions to reduce the total cost of a high severity pandemic is due to the largest component of the overall cost being productivity losses arising from deaths. This is illustrated in Figure 4 which shows the cost components for each intervention. It can be seen that the majority of the cost for an unmitigated pandemic of severity category 4 and 5 is due to death-related productivity losses (shown in purple). Although highly effective interventions incur large intervention-related productivity losses (shown in green), for high severity pandemics these intervention costs are more than outweighed by the reduction in medical costs and death-related productivity losses.

The most costly intervention considered (i.e. which still reduced the attack rate by at least 50%) is continuous school closure combined with continuous workforce reduction, which costs $4,804 per person.

### Low severity pandemics

For low severity pandemics (in category 1, having CFR < = 0.1%) the intervention strategy with the lowest total cost considered is 8 weeks school closure combined with antiviral treatment and prophylaxis, costing $374 per person which represents a net saving of $67 per person compared to no intervention. However, this strategy is not as effective as other intervention strategies, reducing the attack rate to only 15%.

The most effective intervention (combined continuous school closure, community contact reduction, and antiviral treatment and household prophylaxis), which reduces the attack rate to 4.6%, costs $416 per person, a net benefit of $25 per person compared to no intervention. Figure 4 shows that for category 1 and 2 pandemics, although highly effective intervention measures reduce medical costs and death-related productivity losses, they incur larger costs due to intervention-related lost productivity.

The most costly intervention considered is continuous school closure combined with continuous workforce reduction, which costs $1,217 per person, a net cost of $776 per person compared to no intervention. This is due to the large cost associated with 50% workforce absenteeism.

### Non-pharmaceutical interventions

An important subset of intervention strategies are those consisting of purely social distancing interventions. In the case that antiviral drugs are unavailable or ineffective, only these non-pharmaceutical interventions strategies will be available. The most effective non-pharmaceutical strategy is the continuous application of the three social distancing interventions, school closure, workforce reductions, and community contact reduction, which reduces the attack rate to 6%. This intervention has a total cost ranging from $1,116 to $2,603 per person for severity categories ranging from 1 to 5 respectively.

The least costly non-pharmaceutical strategy omits workforce reduction, resulting in a slightly higher attack rate of 7%. This intervention has a total cost ranging from $447 to $2,275 per person for severity categories ranging from 1 to 5 respectively.

### Results without death-related productivity losses

The costing model used for this analysis includes future productivity losses from deaths caused by the pandemic. This long-term cost is often not included in cost-utility analyses. The inclusion of death-related productivity losses greatly increases the total costs of severe pandemics. However, even if these costs are not included, medical costs (due to hospitalisation and ICU usage) play a similar, although less extreme, role. If long-term productivity losses due to death are not included in the costing model, the total cost of the pandemic is not surprisingly lower. However the effectiveness and *relative total costs* of intervention strategies – that is, the ranking of intervention strategies by total cost - remains the same whether or not death-related productivity losses are included (Spearman’s rank correlation coefficient r = 0.95, p = 0.006 for a null hypothesis that rankings are uncorrelated). Full cost results of an alternate analysis that omits death-related productivity losses is contained in an additional file accompanying this paper (Additional file 1), and is summarised below.

For category 5, when death-related productivity losses are not included the total cost of intervention strategies ranges from $559 to $1,711. This range is much smaller than if death-related productivity losses are included, in which case total cost ranges from $1,799 to $4,804. For lower severity pandemics with lower case fatality ratios, the contribution of death-related productivity losses is naturally smaller. For category 1, when death-related productivity losses are not included total cost ranges from $314 to $1,089; with death-related productivity losses the range is $365 to $1,217.

If death-related productivity losses are not included, social distancing and illness costs dominate the total cost of each intervention strategy for low severity pandemics, while health care costs dominate the cost profile for high severity pandemics.

### Sensitivity analyses

Sensitivity analyses were conducted to examine the extent to which these results depend upon uncertain model parameters that may impact on the cost or effectiveness of interventions. The methodology adopted was to identify assumptions and model parameters known to have an effect on intervention outcomes, taken from previous studies with this simulation model [4,5,10,11,37,52], and to perform univariate analyses on each, examining parameter values both significantly higher and lower than the baseline values. Alternative parameter settings were analysed for transmissibility (as characterised by the basic reproduction number R_0_), voluntary household isolation of symptomatic individuals, antiviral efficacy, compliance to home isolation during school closure, degree of workforce reduction, and degree of community contact reduction.

A common finding across all sensitivity analyses was that alternative parameter settings that rendered interventions less effective resulted in strategies that not only had higher attack rates, but also had higher total pandemic costs, with this effect being most pronounced for pandemics of high severity.

Further details and results of the sensitivity analysis can be found in an additional file accompanying this paper (Additional file 1).

## Discussion

The need for an unambiguous, extended definition of severity has been noted in the World Health Organization report on the handling of the 2009 pandemic [59], which highlights the impact pandemic severity has on health care provision and associated costs. In the absence of such definitions, an extended severity metric is presented. This extends the case fatality ratio (CFR) severity scale devised by the CDC [42], with hospitalisation and intensive care unit (ICU) data collected in Australia during the 2009 pandemic. These data have been used to generate a more extensive notion of pandemic severity, relating actual age-specific attack rates with age-specific hospitalisation and mortality rates, thereby contributing to the realism of both the simulation model and the economic analysis. This pandemic severity scale together with a pandemic spread simulation model allows the calculation of the total cost of a pandemic, and to estimate the relative magnitude of all the factors that contribute to the pandemic cost, including not only pharmaceutical and medical costs, but also productivity losses due to absenteeism and death.

The severity of a future pandemic is shown to have a major impact on the overall cost to a nation. Unsurprisingly, high severity pandemics are shown to be significantly more costly than those of low severity, using a costing methodology which includes costs arising from losses to the economy due to death, in addition to intervention and healthcare costs. A key finding of this study is that at high severity categories, total pandemic costs are dominated by hospitalization costs and productivity losses due to death, while at low severities costs are dominated by productivity losses due to social distancing interventions resulting from closed schools and workplaces.

Consequently, findings indicate that at high severity, the interventions that are the most effective also have the lowest total cost. Highly effective interventions greatly reduce the attack rate and consequently the number of deaths, which in turn reduces productivity losses due to death. Although highly effective interventions incur significant intervention-related productivity losses, for severe pandemics having high CFR, these intervention costs are more than compensated for by the reduction in death-related productivity losses, resulting in lower overall costs. Conversely, for low severity pandemics, although highly effective intervention measures do reduce medical costs and death-related productivity losses, these savings can be smaller than costs incurred due to intervention-related lost productivity, resulting in total costs that are higher than the unmitigated baseline.

Antiviral strategies alone are shown to be ineffective in reducing the attack rate by at least 50%. However, the addition of antiviral case treatment and household prophylaxis to any social distancing strategy always resulted in lower attack rates and lower total costs when compared to purely social distancing interventions. The cost of all antiviral interventions constitutes a small fraction of total pandemic costs, and these costs are outweighed by both the healthcare costs prevented, and productivity gained, by their use in preventing illness and death.

### Study limitations

It should be noted that the lowest severity category considered, pandemic category 1, has a CFR of 0.1% which is at the upper end of CFR estimates for the 2009 pandemic, which has been estimated to have a CFR of between 0.018% and 0.159% [51]. Thus, the cost results are not directly applicable to the 2009 pandemic.

Vaccination has been deliberately omitted from this study. The effectiveness and cost effectiveness of vaccination will depend crucially on the timing of the availability of the vaccine relative to the arrival of the pandemic in the community – vaccination cannot be plausibly modelled without considering this delay, and how it interacts with the timing of introduction and relaxation of other, rapidly activated interventions. The examination these timing issues for realistic pandemic scenarios that include both vaccination and social distancing / antiviral interventions is an important avenue for future work. As they stand, the results of this study, specifically the “continuous” duration social distancing strategies, can be considered to be models of interim interventions to be used prior to a vaccination campaign.

The results are based on the community structure, demographics and healthcare system of a combined rural and urban Australian community, and as such may not be applicable to developing world communities with different population or healthcare characteristics. Although the cost and effectiveness results are directly applicable to pandemic interventions in a small community of 30,000 individuals, we expect that the per-capita costs and final attack rate percentages derived in this study can be extended to larger populations with similar demographics, provided a number of conditions are met. For the results to be generalisable, it needs to be assumed that communities making up the larger population implement the same intervention strategies, and instigate interventions upon the arrival of the pandemic in the local community (according to the criteria described in the Methods section). The assumption is also made that there are no travel restrictions between communities. It should be noted that the single-community epidemic results do not predict the overall timing of the pandemic in the larger population.

### Related research

The simulation model used in this study has been used in previous studies to examine various aspects of social distancing and pharmaceutical (antiviral and vaccine) pandemic influenza interventions [4,5,10,11,37,52]. This simulation model shares characteristics with other individual-based pandemic influenza simulation models that have been employed at a variety of scales, including small communities [7,10,13,38,60,61], cities [8,62], countries [6,23,34,63] and whole continents [64].

Several related studies which also used individual-based simulation models of influenza spread coupled with costing models are those of those of Sander et al., Perlroth et al., Brown et al., and Andradottir et al. [45,46,57,65]. The current study extends upon the scope of these studies in several ways: five gradations of pandemic severity are considered, more combinations of interventions are considered, social distancing interventions of varying durations are considered, and probabilities of severe health outcomes for each severity category are based on fatality, hospitalization and ICU usage data as observed from the 2009 pandemic. Also in contrast with those models, we have chosen to include a cost component arising from productivity loss due to death, though a similar costing without death-related productivity losses has been included in (Additional file 1).

For a pandemic with very low severity, with a CFR consistent with mild seasonal influenza, and that of the 2009 pandemic, previous results with the simulation and costing model used for this paper coincide with the studies mentioned above [52]. Specifically, they showed that antiviral treatment and prophylaxis were effective in reducing the attack rate and had a low or negative incremental cost, and that adding continual school closure further decreased attack rates, but significantly increased total cost.

For high severity pandemics the inclusion of productivity loss following death, as presented in this study, leads to a markedly different assessment of total costs when compared to the two studies quoted above that considered severe pandemics [45,46]. For example, Perlroth et al. found that the incremental cost of adding continuous school closure to an antiviral strategy was always positive, even for pandemics with high transmissibility (R_0_ = 2.1) and a CFR of up to 2%, meaning that adding school closure always increased total costs. Similarly Sander et al. found that the addition of continuous school closure to an extended antiviral strategy also increased total costs, including pandemics with a 5% CFR. In contrast, we found that adding continuous school closure to an extended prophylaxis strategy reduced total costs where the CFR was 0.25% or greater (i.e. category 2 and above), for a pandemic with R_0_ = 1.8.

The study of Smith et al. estimated the economic impact of pandemic influenza on gross domestic product for a range of transmissibility and severity values [66]. Consistent with our study was the finding that at low severity the largest economic impacts of a pandemic would be due to school closure (effective but costly) and workplace absenteeism (largely ineffective and costly). Like the other two studies mentioned above, the study of Smith et al. did not include future productivity losses due to death. As a result, in contrast to our findings, they did not find that, for severe pandemics, the high short-term costs of rigorous social distancing interventions were outweighed by future productivity of people whose lives were saved by the intervention.

In this study we considered the case of a pandemic that infects a significant proportion of the population, and thus incurs significant direct costs stemming from medical costs and productivity losses. However, in the case of a pandemic perceived by the public to be severe, there are likely to be additional indirect macroeconomic impacts caused by disruption of trade and tourism, consumer demand and supply, and investor confidence [67,68]. In the case of a pandemic of high severity (i.e. high case fatality ratio) but low transmissibility, these indirect effects and their resulting societal costs may constitute the main economic impact of the pandemic, an effect seen with the SARS outbreak in 2003 [67].

## Conclusions

The results of this study are relevant to public health authorities, both in the revision of pandemic preparedness plans, and for decision-making during an emerging influenza pandemic. Recent modelling research has shown that combinations of social distancing and pharmaceutical interventions may be highly effective in reducing the attack rate of a future pandemic [5,6,8,9,12,13,23,34],[62]. Public health authorities are aware that rigorous social distancing measures, which were used successfully in some cities during the 1918 pandemic [69,70], when pharmaceutical measures were unavailable, would be highly unpopular due to resulting societal disruption, and costly due to associated productivity losses [66]. The results of this study give guidance as to the pandemic characteristics which warrant the use of such interventions.

The results highlight the importance of understanding the severity of an emergent pandemic as soon as possible, as this gives guidance as to which intervention strategy to adopt. In the likely situation where the severity of an emerging pandemic is initially unknown (but is suspected to be greater than that of seasonal influenza), the results indicate that the most appropriate intervention strategy is to instigate school closure and community contact reduction, combined with antiviral drug treatment and household prophylaxis, as soon as transmission has been confirmed in the community. If severity is determined to be low, public health authorities may consider relaxing social distancing measures. In the case of a category 1 pandemic (CFR approximately 0.1%), little is lost by the early imposition and subsequent relaxation of social distancing interventions: results indicate that even if schools are closed for 8 weeks while severity is being determined, the total cost of the pandemic is lower than if no interventions had been enacted. If severity is determined to be high, extending the duration of social distancing interventions results in both net savings to society and reduction in mortality.

## Competing interests

GJM has received a travel grant from GlaxoSmithKline to attend an expert meeting in Boston, USA; MJP has received travel grants from GlaxoSmithKline and Wyeth to attend expert meetings in Reykjavik, Iceland, Boston, USA and Istanbul, Turkey. JKK and NH have no potential competing interests.

## Authors’ contributions

GJM and MJP designed the study. GJM, JKK and NH designed the computer model. NH and JKK carried out the computer simulations and analysis. Data analyses were performed by NH and JKK under the supervision of GJM. GJM, JKK and NH drafted the manuscript. All authors commented on drafts and contributed to the final version. All authors read and approved the final manuscript.

## Pre-publication history

The pre-publication history for this paper can be accessed here:

http://www.biomedcentral.com/1471-2458/13/211/prepub

## Supplementary Material

Additional file 1 **Additional Results and Sensitivity Analyses.** “Milne2013PandemicCostAdditionalFile1.doc”. Click here for file
