# Supplementary material for: Economic analysis of pandemic influenza mitigation strategies for five pandemic severity categories
Source: BMC Public Health. 2013 Mar 8;13:211. doi: 10.1186/1471-2458-13-211 (PMC3606600; doi:10.1186/1471-2458-13-211)
Supplement: Additional file 1 — Additional Results and Sensitivity Analyses. “Milne2013PandemicCostAdditionalFile1.doc”. [file 1471-2458-13-211-S1.doc]

# Economic Analysis of Pandemic Mitigation Strategies for Five Pandemic Severity Categories – Additional File 1

## Results Including Extended Antiviral Prophylaxis

**Table S0** Intervention costs and cost effectiveness.

| **Intervention Strategy** | **AR (%)** | **Cat 1, CFR=0.1%** | **Cat 2, CFR=0.25%** | **Cat 3, CFR=0.75%** | **Cat 4, CFR=1.5%** | **Cat 5, CFR=2.5%** |
| --- | --- | --- | --- | --- | --- | --- |
| **no intervention** | **32.4** | **$441** | **$943** | **$2,649** | **$5,175** | **$8,550** |
| **SC 2 wks + T+H+E** | **15.4** | **$345** | **$585** | **$1,402** | **$2,611** | **$4,226** |
| ***SC cont + WR cont** | **15.7** | **$1,217** | **$1,439** | **$2,194** | **$3,311** | **$4,804** |
| **SC 8 + WR 4 + CCR 4 + T+H** | **14.9** | **$539** | **$757** | **$1,499** | **$2,596** | **$4,062** |
| **SC 8 wks + T+H** | **14.5** | **$374** | **$582** | **$1,288** | **$2,334** | **$3,732** |
| ***SC cont + CCR 4** | **14.5** | **$518** | **$722** | **$1,419** | **$2,449** | **$3,826** |
| ***SC cont + WR 4 + CCR 4** | **13.2** | **$654** | **$854** | **$1,533** | **$2,539** | **$3,882** |
| **SC 8 wks + T+H+E** | **12.9** | **$382** | **$566** | **$1,191** | **$2,116** | **$3,352** |
| **SC 8 + WR 4 + CCR 4 + T+H+E** | **11.2** | **$525** | **$700** | **$1,296** | **$2,176** | **$3,353** |
| **SC cont + T+H** | **9.2** | **$489** | **$629** | **$1,104** | **$1,808** | **$2,748** |
| **SC cont + T+H+E** | **8.0** | **$493** | **$614** | **$1,028** | **$1,639** | **$2,457** |
| ***SC cont + CCR cont** | **7.4** | **$447** | **$560** | **$945** | **$1,514** | **$2,275** |
| **SC cont + WR 4 + CCR 4 + T+H+E** | **7.1** | **$587** | **$696** | **$1,069** | **$1,621** | **$2,359** |
| **SC cont + WR 4 + CCR 4 + T+H** | **7.9** | **$585** | **$691** | **$1,052** | **$1,585** | **$2,298** |
| ***SC cont + WR cont + CCR cont** | **6.0** | **$1,116** | **$1,208** | **$1,521** | **$1,984** | **$2,603** |
| **SC cont + CCR cont + T+H+E** | **6.7** | **$365** | **$454** | **$756** | **$1,202** | **$1,799** |
| **SC cont + WR cont + CCR cont + T+H+E** | **6.7** | **$1,027** | **$1,118** | **$1,426** | **$1,883** | **$2,494** |
| **SC cont + CCR cont + T+H** | **5.7** | **$416** | **$488** | **$734** | **$1,098** | **$1,584** |
| **SC cont + WR cont + CCR cont + T+H** | **5.6** | **$1,083** | **$1,155** | **$1,401** | **$1,764** | **$2,249** |

Cost of pandemic shown as total cost for each intervention strategy and each severity category. Costs expressed as dollars (US) per member of population. Values are for pandemic with unmitigated transmissibility of R_0_ = 1.8. Interventions abbreviated as: SC – school closure; CCR – 50% community contact reduction; WR – 50% workforce reduction; 4, 8 – intervention duration in weeks; cont – continuous duration; T – antiviral treatment of diagnosed symptomatic cases; H – antiviral prophylaxis of household members of diagnosed symptomatic cases, E – antiviral prophylaxis of school class or workplace contacts of diagnosed symptomatic cases. Pure social distancing interventions marked by *.

## Death-related Productivity Losses

The costing model used for this analysis includes future productivity losses from deaths caused by the pandemic. The inclusion of death-related productivity losses greatly increases the total costs of severe pandemics. We repeated our cost analyses for all interventions and severity categories – Figures S1, S2 and S3, and Table S1 below give results equivalent to Figures 3 and 4 and Table 3 in the main paper, but without the inclusion of death-related productivity costs.

**Figure S1.** Total pandemic cost for each severity category. Costs shown by colour coded columns according to pandemic severity, with cost per person in community shown on left axis. Intervention strategies are listed on horizontal axis. Attack rates (AR) for each strategy appear with each strategy label. Values are for a pandemic with unmitigated transmissibility of R = 1·8. Interventions are abbreviated as: SC – school closure; CCR – 50% community contact reduction; WR – 50% workforce reduction; 4, 8 – intervention duration in weeks; cont – continuous duration; T – antiviral treatment of diagnosed symptomatic cases; H – antiviral prophylaxis of household members of diagnosed symptomatic cases, E – antiviral prophylaxis of school class or workplace contacts of diagnosed symptomatic cases.

**Figure S2.** Breakdown of pandemic costs shown as horizontal bar, for each intervention strategy and each severity category. Coloured segments of each bar represent cost components as follows: (blue) health care; (red) antiviral drugs, including dispensing costs; (green) productivity losses due to illness and social distancing interventions; (purple) productivity losses due to deaths. Values are for a pandemic with unmitigated transmissibility of R = 1.8. Interventions are abbreviated as for Figure S1.

**Table S1 Intervention costs and cost effectiveness without productivity losses due to death.**

| **Intervention Strategy** | **AR (%)** | **Cat 1, CFR=0.1%** | **Cat 2, CFR=0.25%** | **Cat 3, CFR=0.75%** | **Cat 4, CFR=1.5%** | **Cat 5, CFR=2.5%** |
| --- | --- | --- | --- | --- | --- | --- |
|  |  |  |  |  |  |  |
| **no intervention** | **32** | $151 | $236 | $531 | $963 | $1,538 |
| **SC 2 wks + T+H+E** | **15** | $206 | $247 | $387 | $594 | $868 |
| ***SC cont + WR cont** | **15** | $1,089 | $1,127 | $1,259 | $1,453 | $1,711 |
| **SC 8 + WR 4 + CCR 4 + T+H** | **14** | $413 | $450 | $577 | $765 | $1,014 |
| **SC 8 wks + T+H** | **14** | $254 | $289 | $412 | $592 | $832 |
| ***SC cont + CCR 4** | **14** | $400 | $435 | $558 | $737 | $977 |
| ***SC cont + WR 4 + CCR 4** | **13** | $539 | $574 | $693 | $868 | $1,101 |
| **SC 8 wks + T+H+E** | **12** | $276 | $308 | $416 | $575 | $787 |
| **SC 8 + WR 4 + CCR 4 + T+H+E** | **11** | $424 | $454 | $557 | $707 | $908 |
| **SC cont + T+H** | **9** | $408 | $433 | $516 | $639 | $802 |
| **SC cont + T+H+E** | **8** | $423 | $444 | $517 | $623 | $766 |
| ***SC cont + CCR cont** | **7** | $382 | $402 | $469 | $568 | $700 |
| **SC cont + WR 4 + CCR 4 + T+H+E** | **7** | $523 | $542 | $607 | $702 | $829 |
| **SC cont + WR 4 + CCR 4 + T+H** | **7** | $524 | $542 | $605 | $697 | $820 |
| ***SC cont + WR cont + CCR cont** | **6** | $1,063 | $1,079 | $1,133 | $1,213 | $1,319 |
| **SC cont + CCR cont + T+H+E** | **6** | $314 | $329 | $381 | $457 | $559 |
| **SC cont + WR cont + CCR cont + T+H+E** | **6** | $974 | $989 | $1,042 | $1,119 | $1,222 |
| **SC cont + CCR cont + T+H** | **5** | $374 | $387 | $429 | $492 | $576 |
| **SC cont + WR cont + CCR cont + T+H** | **5** | $1,041 | $1,054 | $1,096 | $1,157 | $1,240 |

Cost of pandemic shown as total cost for each intervention strategy and each severity category. Costs expressed as dollars (US) per member of population. Values are for pandemic with unmitigated transmissibility of R = 1.8. Interventions abbreviated as: SC – school closure; CCR – 50% community contact reduction; WR – 50% workforce reduction; 4, 8 – intervention duration in weeks; cont – continuous duration; T – antiviral treatment of diagnosed symptomatic cases; H – antiviral prophylaxis of household members of diagnosed symptomatic cases, E – antiviral prophylaxis of school class or workplace contacts of diagnosed symptomatic cases. Pure social distancing interventions marked by *.

## Sensitivity Analyses

## Transmissibility

We further examined the total cost and effectiveness for all intervention strategies and severity categories for pandemic strains having unmitigated transmission characteristics both lower and higher than our primary assumption of reproduction number R_0_=1.8, with a low transmission setting having R_0_=1.5 and high transmission R_0_=2.5. A pandemic with a reproduction number of 1.5 corresponds to some estimations of the effective reproduction number of the 2009 pandemic while a reproduction number of 2.5 corresponds to an upper bound on estimates of what may have occurred in the 1918 pandemic, with most estimates being in the range 1.8-2.2 .

Figures S3A and S3B show attack rates and total costs for R_0_=1.5 and R=2.5 respectively. Only intervention strategies that reduce the attack rate by at least 50% are presented. The effectiveness of interventions in reducing the illness attack rate is directly related to disease transmissibility, as shown by prior modelling studies . Thus in Figure S3A the low transmissibility scenario includes 22 effective intervention strategies, while the high transmissibility scenario of Figure S3B considers 10 intervention strategies.

Comparing intervention cost results for R_0_=1.5 (Figure S3A) with that for R_0_=1.8 (Figure 3) it is apparent that total costs for low severity levels (category 2 and below) are comparable; total costs are principally composed of intervention-related productivity losses which do not vary with transmissibility. At the highest severity levels (category 3 and above) some of the R_0_=1.8 total costs are higher due to medical and death-related productivity losses. Within each severity category, the overall pattern of cost and cost effectiveness is similar when comparing the eighteen strategies present in Figure 3 with the same strategy subset in Figure S3A.

Comparing the effective interventions in Figure S3B (R_0_=2.5) with the same interventions in Figure 3 (R_0_=1.8) both the magnitude and relative effectiveness of interventions differ. For low severity levels intervention cost results are similar, but for higher severity categories both the total cost and the spread of costs across intervention strategies is greater for the higher transmission R_0_=2.5 situation.


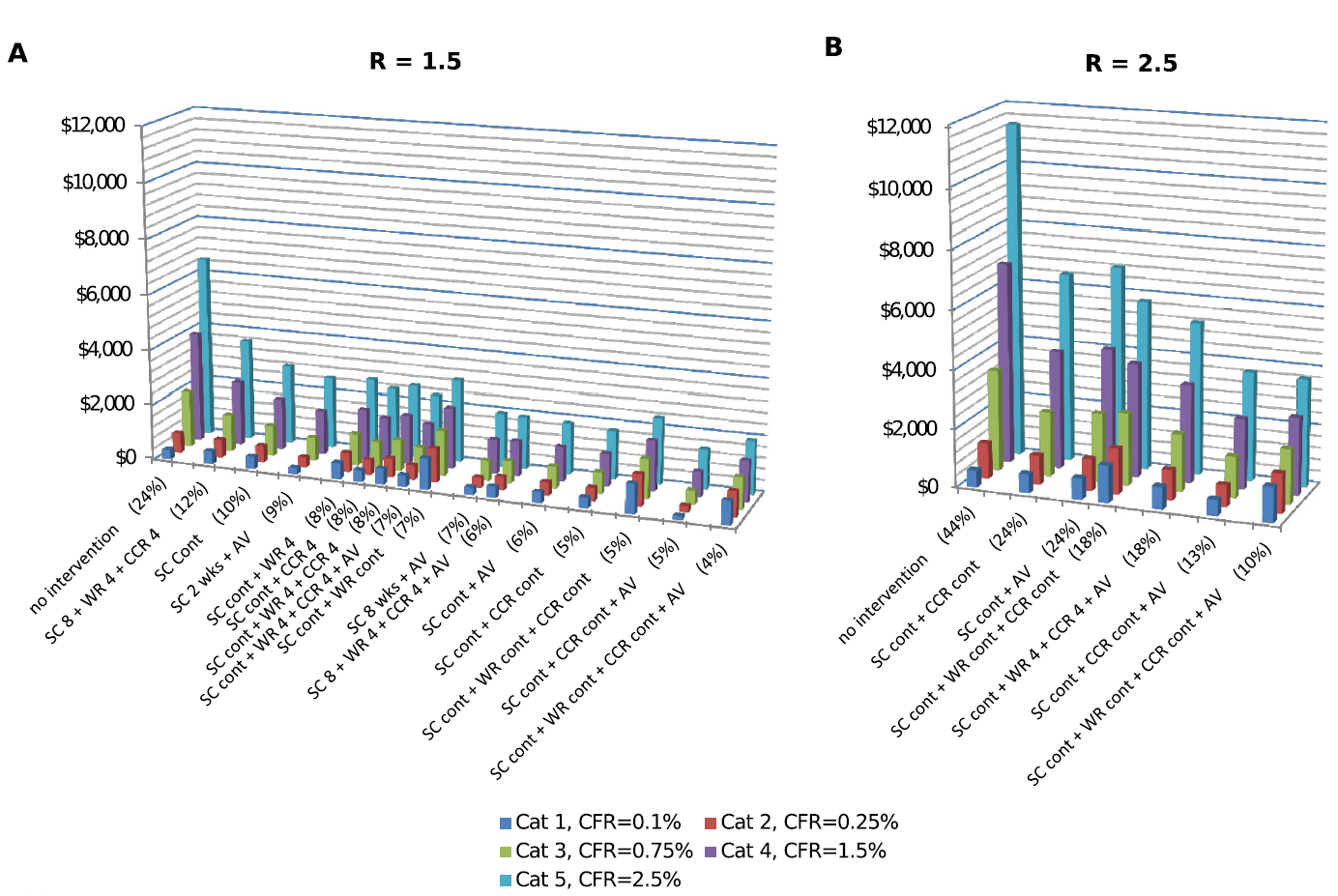


## Figure S3. Total pandemic cost for each severity category, left panel (A) for reproduction number R_0_ = 1.5, right panel (B) for reproduction number R_0_ = 2.5 Costs shown by colour coded columns according to pandemic severity, with cost per person in community shown on left axis. Intervention strategies are listed on horizontal axis. Attack rates (AR) for each strategy appear with each strategy label. Values are for a pandemic with unmitigated transmissibility of R_0_ = 1.8. Interventions abbreviated as: SC – school closure; CCR – 50% community contact reduction; WR – 50% workforce reduction; 4, 8 – intervention duration in weeks; cont – continuous duration; AV – antiviral treatment of diagnosed symptomatic cases and antiviral prophylaxis of household members of diagnosed symptomatic cases.

## Voluntary Household Isolation of Symptomatic Individuals

An underlying assumption of the simulation model is that 50% adults (and 90% of children) who become infected and symptomatic will withdraw to their household for the duration of their illness. Sensitivity analysis results for this parameter are given in Table S2. If 25% of adults self-isolate rather than 50%, the no intervention final attack rate is 39% rather than 32%. In addition, intervention strategies are also less effective in reducing the attack rate. This is true for all severity categories.

These results suggest that the total cost to society of individuals working while ill is higher than if they stayed at home. Even assuming that symptomatic individuals are fully productive, the extra productivity of ill individuals is outweighed by medical costs and lost productivity due to the larger epidemic caused by additional infection transmission due to sick workers. This is most pronounced with less effective intervention strategies. Under the original assumption of 50% voluntary isolation, a strategy of two weeks school closure combined with antiviral treatment and prophylaxis reduces the final attack rate by 17 percentage points (32% to 15%), costs $345 per person. If voluntary isolation is only 25%, the attack rate is reduced by 13 percentage points (from 39% to 26%), costing $489 per person. If on the other hand 75% of adults self-isolate rather than 50%, the no intervention final attack rate is 25% rather than 32%. In this case total costs are slightly lower. Some productivity is lost due to sick individuals staying at home who would otherwise have worked, but because increased isolation results in a smaller epidemic, the total cost is lower.

## Other Model Parameters

Sensitivity analyses were also conducted to assess key parameters related to each of the intervention strategies. Alternative higher and lower parameter values for antiviral efficacy, compliance to home isolation during school closure, degree of workforce reduction, and degree of community contact reduction were examined and the effect which these parameter settings have on the resulting attack rate and total cost of interventions were determined. Results of these sensitivity analyses for severity categories 1 and 5 are presented in Tables S2 – S5.

An outcome of the sensitivity analyses is the observation that intervention strategies that combine multiple interventions are much less sensitive to parameter changes than interventions they rely on a single type of intervention. This is because if one intervention in a combination strategy is less (or more) effective than anticipated, the total effectiveness and cost of the intervention strategy still includes the contributions of the other interventions.

If the efficacy of antiviral drugs is half that of the efficacy estimated from trials (lowering infectiousness by 33% instead of 66%, and lowering susceptibility by 42% instead of 85%), strategies which rely wholly or primarily on antiviral interventions are less effective, more costly and less cost effective. However, even at this lower efficacy the addition of antiviral treatment and household prophylaxis to rigorous social distancing interventions results in an additional attack rate reduction of 2 percentage points and a lower total cost. If the efficacy of antiviral drugs is higher than estimated (lowering infectiousness and susceptibility by 80% and 95% respectively) antiviral-only strategies are slightly more effective. Sensitivity analysis results for antiviral efficacy are given in Table S3.

Previous studies have identified the contact behaviour of school children when school closures are in effect as being critical in determining the effectiveness of school closure . The underlying assumption of the simulation model is that when school closure is in effect students have no potentially infective contact with their school class group. If it is instead assumed that only 75% (or 50%) of school students maintain strict household isolation (and that the others meet class group members also breaking household isolation), strategies consisting of school closure only are less effective and more costly. If school closure is combined with other interventions, costs rise to a much smaller degree. School closure sensitivity analysis results are given in Table S4.

Workforce reduction is the least cost effective of all the modelled interventions. The inclusion of less rigorous workforce reduction (where workers stay at home 25% rather than 50% of work days) generally makes intervention strategies slightly less effective but also less costly; conversely, more rigorous workforce reduction (household isolation on 75% or work days) results in intervention strategies that are slightly more effective but much more costly. Workforce reduction sensitivity analysis results are given in Table S5.

The degree to which a public health policy of community contact reduction will actually reduce the number of potentially infective contacts between individuals is difficult to estimate. We have assumed a 50% reduction in “random” or “untraceable” contacts when a community contact reduction intervention is in effect. For a strategy of long term community contact reduction and school closure, achieving only a 25% rather than 50% reduction in community contact results a final attack rate of 12% compared to 7%, and lower total costs, especially for severe pandemics. Community contact reduction sensitivity analysis results are given in Table S6.

**Table S2** - Sensitivity to probability of voluntary isolation given symptoms

|  | **Cat 1 (CFR – 0.1%)** | | | | |
| --- | --- | --- | --- | --- | --- |
| **Voluntary isolation probability** | ***no int.*** | ***SC 2 wks + T + H + E*** | ***SC Cont + T + H + E*** | ***SC Cont + WR Cont + CCR Cont*** | ***SC Cont + CCR Cont + T + H*** |
| **0.25** adult (**0.5** child) | 39% | 26% | 16% | 14% | 9% |
|  | $512 | $489 | $619 | $1214 | $492 |
| *Baseline*  **0.5** adult (**0.9** child) | 32% | 15% | 8% | 6% | 5% |
|  | $441 | $345 | $493 | $1116 | $416 |
| **0.75** adult (**1.0** child) | 27% | 13% | 6% | 5% | 5% |
|  | $380 | $307 | $454 | $1099 | $384 |

|  | **Cat 5 (CFR – 2.5%)** | | | | |
| --- | --- | --- | --- | --- | --- |
| **Voluntary isolation probability** | ***no int.*** | ***SC 2 wks + T + H + E*** | ***SC Cont + T + H + E*** | ***SC Cont + WR Cont + CCR Cont*** | ***SC Cont + CCR Cont + T + H*** |
| **0.25** adult (**0.5** child) | 39% | 26% | 16% | 14% | 9% |
|  | $10233 | $6905 | $4399 | $4602 | $2605 |
| *Baseline*  **0.5** adult (**0.9** child) | 32% | 15% | 8% | 6% | 5% |
|  | $8550 | $4226 | $2457 | $2603 | $1584 |
| **0.75** adult (**1.0** child) | 27% | 13% | 6% | 5% | 5% |
|  | $7158 | $3569 | $1963 | $2353 | $1658 |

Final illness attack rates (%) and total costs (bold text) are given for 4 intervention strategies (columns), for the baseline assumption that 50% of symptomatic adults (and 90% of symptomatic children) would stay at home, and for 2 alternate parameter values. Results are presented for category 1 (upper table) and category 5 (lower table). Interventions are abbreviated as for Table 2 : SC – school closure; CCR – 50% community contact reduction; WR – 50% workforce reduction; 4, 8 – intervention duration in weeks; cont – continuous duration; AV – antiviral treatment of diagnosed symptomatic cases and antiviral prophylaxis of household members of diagnosed symptomatic cases

**Table S3** Antiviral drug efficacy sensitivity analysis

|  | **Cat 1 (CFR – 0.1%)** | | | | | | |
| --- | --- | --- | --- | --- | --- | --- | --- |
| ***Antiviral efficacy:***  ***infectiousness reduction***  ***(susceptibility reduction)*** | ***no int.*** | ***T+H*** | ***T+H+E*** | ***SC 2 wks + T+H+E*** | ***SC Cont + T + H + E*** | ***SC Cont + WR Cont + CCR Cont + T+H+E*** | ***SC Cont + CCR Cont + T + H*** |
| Antiviral efficacy  **11% (14%)** | **32%** | **28%** | **27%** | **23%** | **12%** | **6%** | **5%** |
|  | $441 | $454 | $473 | $455 | $561 | $1,128 | $435 |
| Antiviral efficacy  **33% (42%)** | **32%** | **26%** | **23%** | **20%** | **10%** | **6%** | **5%** |
|  | $441 | $424 | $421 | $409 | $527 | $1,117 | $428 |
| *Baseline* Antiviral efficacy  **66% (85%)** | **32%** | **23%** | **19%** | **15%** | **8%** | **6%** | **5%** |
|  | $441 | $383 | $355 | $345 | $493 | $1,027 | $416 |
| Antiviral efficacy  **80% (95%)** | **32%** | **22%** | **18%** | **14%** | **7%** | **6%** | **5%** |
|  | $441 | $373 | $341 | $329 | $480 | $988 | $412 |

|  | **Cat 5 (CFR – 2.5%)** | | | | | | |
| --- | --- | --- | --- | --- | --- | --- | --- |
| ***Antiviral efficacy:***  ***infectiousness reduction***  ***(susceptibility reduction)*** | ***no int.*** | ***T + H*** | ***T+H+E*** | ***SC 2 wks + T+H+E*** | ***SC Cont + T + H + E*** | ***SC Cont + WR Cont + CCR Cont +T+H+E*** | ***SC Cont + CCR Cont + T + H*** |
| Antiviral efficacy  **11% (14%)** | **32%** | **28%** | **27%** | **23%** | **12%** | **6%** | **5%** |
|  | $8,550 | $7,502 | $7,326 | $6,252 | $3,583 | $2,398 | $1,783 |
| Antiviral efficacy  **33% (42%)** | **32%** | **26%** | **23%** | **20%** | **10%** | **6%** | **5%** |
|  | $8,550 | $6,946 | $6,332 | $5,392 | $3,012 | $2,248 | $1,687 |
| *Baseline* Antiviral efficacy  **66% (85%)** | **32%** | **23%** | **19%** | **15%** | **8%** | **6%** | **5%** |
|  | $8,550 | $6,139 | $5,080 | $4,226 | $2,457 | $2,494 | $1,584 |
| Antiviral efficacy  **80% (95%)** | **32%** | **22%** | **18%** | **14%** | **7%** | **6%** | **5%** |
|  | $8,550 | $5,944 | $4,813 | $3,950 | $2,293 | $2,525 | $1,570 |

Final attack rates (bold face, above shaded rows) and total costs (shaded rows) are given for 7 intervention strategies (columns), for the baseline best-estimate antiviral efficacy values and for 3 alternative efficacy parameter settings. Results are presented for category 1 (upper table) and category 5 (lower table). Interventions are abbreviated as for Figure S1.

**Table S4** School closure home isolation compliance sensitivity analysis

|  | **Cat 1 (CFR – 0.1%)** | | | | | | |
| --- | --- | --- | --- | --- | --- | --- | --- |
| ***School Closure home isolation compliance*** | ***no int.*** | ***SC 2 wks*** | ***SC Cont*** | ***SC Cont + T + H***  ***+ E*** | ***SC Cont + WR Cont + CCR Cont*** | ***SC Cont + WR Cont + CCR Cont + T + H*** | ***SC Cont + CCR Cont + T + H*** |
| Home isolation Compliance  **50%** | **32%** | **27%** | **23%** | **10%** | **9%** | **5%** | **6%** |
|  | $441 | $412 | $595 | $489 | $1,138 | $1,081 | $399 |
|  | - | $134,338 | $102,393 | $37,437 | $84,484 | $69,715 | $26,079 |
| Home isolation Compliance  **75%** | **32%** | **27%** | **20%** | **8%** | **7%** | **5%** | **5%** |
|  | $441 | $409 | $577 | $484 | $1,116 | $1,081 | $407 |
|  | - | $127,677 | $73,646 | $34,375 | $74,575 | $68,094 | $25,691 |
| *Baseline* Home isolation compliance **100%** | **32%** | **27%** | **18%** | **8%** | **6%** | **5%** | **5%** |
|  | $441 | $411 | $574 | $493 | $1,116 | $1,083 | $416 |
|  | - | $129,859 | $66,197 | $34,527 | $72,972 | $67,740 | $25,957 |

|  | **Cat 5 (CFR – 2.5%)** | | | | | | |
| --- | --- | --- | --- | --- | --- | --- | --- |
| ***School Closure home isolation compliance*** | ***no int.*** | ***SC 2 wks*** | ***SC Cont*** | ***SC Cont + T + H***  ***+ E*** | ***SC Cont + WR Cont + CCR Cont*** | ***SC Cont + WR Cont + CCR Cont + T + H*** | ***SC Cont + CCR Cont + T + H*** |
| Home isolation Compliance  **50%** | **32%** | **27%** | **23%** | **10%** | **9%** | **5%** | **6%** |
|  | $8,550 | $7,208 | $6,240 | $2,964 | $3,397 | $2,449 | $1,877 |
|  | - | $97,147 | $44,429 | $9,380 | $10,437 | $6,531 | $5,073 |
| Home isolation Compliance  **75%** | **32%** | **27%** | **20%** | **8%** | **7%** | **5%** | **5%** |
|  | $8,550 | $7,148 | $5,368 | $2,534 | $2,740 | $2,296 | $1,659 |
|  | - | $92,254 | $28,363 | $7,451 | $7,572 | $5,983 | $4,332 |
| *Baseline* Home isolation compliance **100%** | **32%** | **27%** | **18%** | **8%** | **6%** | **5%** | **5%** |
|  | $8,550 | $7,167 | $5,005 | $2,457 | $2,603 | $2,249 | $1,584 |
|  | - | $93,737 | $23,881 | $7,123 | $7,043 | $5,818 | $4,088 |

Final attack rates (bold face, above shaded rows) and total costs (shaded rows) are given for 7 intervention strategies (columns), for the baseline assumption that 100% of children affected by school closure would comply with household isolation, and for 2 alternate parameter values. Results are presented for category 1 (upper table) and category 5 (lower table). Interventions are abbreviated as for Figure S1.

**Table S5** Workforce reduction sensitivity analysis

|  | **Cat 1 (CFR – 0.1%)** | | | |
| --- | --- | --- | --- | --- |
| ***Workforce Reduction*** | ***no int*** | ***SC Cont + WR Cont*** | ***SC Cont + WR Cont + CCR Cont*** | ***SC Cont + WR Cont + CCR Cont + T+H+E*** |
| Workforce Reduction **25%** | **32%** | **16%** | **7%** | **6%** |
|  | $441 | $887 | $778 | $699 |
| *Baseline*  Workforce Reduction **50%** | **32%** | **15%** | **6%** | **6%** |
|  | $441 | $1,217 | $1,116 | $1,027 |
| Workforce Reduction **75%** | **32%** | **13%** | **5.5%** | **5%** |
|  | $441 | $1,553 | $1,465 | $1,387 |

|  | **Cat 5 (CFR – 2.5%)** | | | |
| --- | --- | --- | --- | --- |
| ***Workforce Reduction*** | ***no int*** | ***SC Cont + WR Cont*** | ***SC Cont + WR Cont + CCR Cont*** | ***SC Cont + WR Cont + CCR Cont + T+H+E*** |
| Workforce Reduction **25%** | **32%** | **16%** | **7%** | **6%** |
|  | $8,550 | $4,810 | $2,471 | $2,126 |
| *Baseline*  Workforce Reduction **50%** | **32%** | **15%** | **6%** | **6%** |
|  | $8,550 | $4,804 | $2,603 | $2,494 |
| Workforce Reduction **75%** | **32%** | **13%** | **5.5%** | **5%** |
|  | $8,550 | $4,772 | $2,831 | $2,749 |

Final attack rates (bold face, above shaded rows) and total costs (shaded rows) are given for 4 intervention strategies (columns), for the baseline assumption that 50% of workers would stay at home while workforce reduction was in effect, and for 2 alternate parameter values. Results are presented for category 1 (upper table) and category 5 (lower table). Interventions are abbreviated as for Figure S1.

**Table S6** Community contact reduction sensitivity analysis

|  | **Cat 1 (CFR – 0.1%)** | | | | |
| --- | --- | --- | --- | --- | --- |
| ***Community Contact Reduction***  ***(CCR)*** | ***no int*** | ***SC Cont + CCR Cont*** | ***SC Cont + WR Cont + CCR Cont*** | ***SC Cont + WR Cont + CCR Cont + T+H+E*** | ***SC Cont + CCR Cont + T + H*** |
| CCR **75%** | **32%** | **5%** | **4%** | **5%** | **5%** |
|  | $441 | $416 | $1,094 | $936 | $332 |
| *Baseline* CCR **50%** | **32%** | **7%** | **6%** | **6%** | **5%** |
|  | $441 | $447 | $1,116 | $1,027 | $416 |
| CCR **25%** | **32%** | **12%** | **10%** | **5%** | **6%** |
|  | $441 | $507 | $1,157 | $1,120 | $445 |
|  | - | $42,640 | $86,625 | $70,850 | $29,205 |

|  | **Cat 5 (CFR – 2.5%)** | | | | |
| --- | --- | --- | --- | --- | --- |
| ***Community Contact Reduction***  ***(CCR)*** | ***no int*** | ***SC Cont + CCR Cont*** | ***SC Cont + WR Cont + CCR Cont*** | ***SC Cont + WR Cont + CCR Cont + T+H+E*** | ***SC Cont + CCR Cont + T + H*** |
| CCR **75%** | **32%** | **5%** | **4%** | **5%** | **5%** |
|  | $8,550 | $1,635 | $2,185 | $2,154 | $1,631 |
| *Baseline* CCR **50%** | **32%** | **7%** | **6%** | **6%** | **5%** |
|  | $8,550 | $2,275 | $2,603 | $2,494 | $1,584 |
| CCR **25%** | **32%** | **12%** | **10%** | **5%** | **6%** |
|  | $8,550 | $3,527 | $3,500 | $2,380 | $1,972 |

Final attack rates (bold face, above shaded rows) and total costs (shaded rows) are given for 4 intervention strategies (columns), for the baseline assumption that individuals would make 50% fewer community contacts while community contact reduction was in effect, and for 2 alternate parameter values. Results are presented for category 1 (upper table) and category 5 (lower table). Interventions are abbreviated as for Figure S1.

## References

1. Fraser C, Christl DA, Cauchemez S, Hanage WP, Van Kerkhove MD, Hollingsworth TD, Griffin J, Baggaley RF, Jenkins HE, Lyons EJ *et al*: **Pandemic potential of a strain of influenza A (H1N1): early findings**. *Science* 2009, **324**:1557-1561.

2. Kelly H, Mercer G, Fielding J, Dowse G, Glass K, Carcione D, Grant KA, Effler PV, Lester RA, Gravenor MB: **Pandemic (H1N1) 2009 influenza community transmission was established in one Australian state when the virus was first identified in North America**. *PLoS ONE* 2010, **5**(6):e11341.

3. Nishiura H, Wilson N, Baker M: **Estimating the reproduction number of the novel influenza A virus (H1N1) in a Southern Hemisphere setting: preliminary estimate in New Zealand**. *Journal of the New Zealand Medical Association* 2009, **122**(1299):73-77.

4. Frost W: **Statistics of influenza morbidity with special reference to certain factors in case incidence and case fatality**. *Public Heath Report* 1920, **35**:584-597.

5. Glezen WP: **Emerging infections: pandemic influenza**. *Epidemiol Rev* 1996, **18**(1):64-76.

6. Milne GJ, Kelso JK, Kelly HA, Huband ST, McVernon J: **A small community model for the transmission of infectious diseases: comparison of school closure as an intervention in individual-based models of an influenza pandemic**. *PLoS ONE* 2008, **3**(12):e4005.

7. Germann TC, Kadau K, Longini IM, Jr., Macken CA: **Mitigation strategies for pandemic influenza in the United States**. *PNAS* 2006, **103**(15):5935-5941.

8. Ferguson NM, Cummings DAT, Fraser C, Cajka JC, Cooley PC, Burke DS: **Strategies for mitigating an influenza pandemic**. *Nature* 2006, **442**:448-452.

9. Glass RJ, Glass LM, Beyeler WE, Min HJ: **Targeted social distancing design for pandemic influenza**. *Emerg Infect Dis* 2006, **12**(11):1671-1681.

10. Davey VJ, Glass RJ, Min HJ, Beyeler WE, Glass LM: **Effective, robust design of community mitigation for pandemic influenza: a systematic examination of proposed US guidance**. *PLoS ONE* 2008, **3**(7):e2606.

11. Yang Y, Longini IM, Jr., Halloran ME: **Design and evaluation of prophylactic interventions using infectious disease incidence data from close contact groups**. *Appl Statist* 2006, **55**:317-330.

12. Moscona A: **Neuraminidase inhibitors for influenza**. *N Engl J Med* 2005, **353**(13):1363-1373.

13. Cauchemez S, Valleron A-J, Boe¨lle P-Y, Flahault A, Ferguson NM: **Estimating the impact of school closure on influenza transmission from Sentinel data**. *Nature Letters* 2008, **452**:750-755.
